# Structure of natural variant transglutaminase 2 reveals molecular basis of gaining stability and higher activity

**DOI:** 10.1371/journal.pone.0204707

**Published:** 2018-10-15

**Authors:** Hyun Ji Ha, Sunghark Kwon, Eui Man Jeong, Chang Min Kim, Ki Baek Lee, In-Gyu Kim, Hyun Ho Park

**Affiliations:** 1 School of Pharmacy, Chung-Ang University, Seoul, South Korea; 2 Department of Biochemistry and Molecular Biology, Seoul National University College of Medicine, Seoul, South Korea; Russian Academy of Medical Sciences, RUSSIAN FEDERATION

## Abstract

Multi-functional transglutaminase 2 (TG2), which possesses protein cross-linking and GTP hydrolysis activities, is involved in various cellular processes, including apoptosis, angiogenesis, wound healing, and neuronal regeneration, and is associated with many human diseases, including inflammatory disease, celiac disease, neurodegenerative disease, diabetes, tissue fibrosis, and cancers. Although most biochemical and cellular studies have been conducted with the TG2 (G224) form, the TG2 (G224V) form has recently emerged as a putative natural variant of TG2. In this study, we characterized the putative natural form of TG2, TG2 (G224V), and through a new crystal structure of TG2 (G224V), we revealed how TG2 (G224V) gained stability and higher Ca^2+^-dependent activity than an artificial variant of TG2 (G224).

## Introduction

Transglutaminase 2 (TG2) is a multi-functional protein that possesses various biological activities, including protein cross-linking activity [1], GTPase activity [2], protein disulfide isomerase activity [3], kinase activity [4], and scaffold activity [5]. Because of its various functions, TG2 is involved in many important cellular processes, including apoptosis [6], angiogenesis [7], wound healing [7], neuronal regeneration[8], and bone development. Failure in the regulation of TG2 activities is associated with many human diseases, including inflammatory disease [9], celiac disease [10], neurodegenerative disease [11], diabetes [12], tissue fibrosis [13], and cancers [14]. It is known that TG2 function differs according to its location in the cell. In the cytosol, TG2 acts as a signal transfer molecule that transmits a receptor signal to an intracellular effector through GTP hydrolysis [15]. When it is secreted into the extracellular environment, TG2 functions as a cross-linking enzyme in the matrix [16]. This protein transamidase activity of TG2 is positively regulated by calcium and negatively regulated by GTP [17].

Eight different TG isoenzymes have been identified in mammals [18]. Enzymes in this family share a high sequence identity and structural similarity, although the function and substrate specificity vary significantly within the family. Human TG2 is 687 amino acids in length, consisting of four domains, an N-terminal β-sandwich domain, catalytic domain, and two C-terminal β-barrel domains [19], and is the most ubiquitous isoform of the TG family. Because TG2 has diverse activities and serves as a target for therapeutic intervention, extensive structural studies have been conducted with it. As a result, nine crystal structures, in complex with guanosine diphosphate (GDP) [19], adenosine triphosphate (ATP) [20], and guanosine triphosphate (GTP) [21], two autoantibody Feb fragments [22, 23], and four covalently bound inhibitors (three have not been published, but deposited on PDB) [24] have been elucidated. Structural studies showed that TG2 undergoes an extraordinarily large conformational change upon activation; closed inactive form and opened active form. Based on structural and biochemical studies, it is believed that extracellular TG2 is maintained in a closed conformation because of GTP (inhibitor) binding. However, in circumstances where Ca^2+^ concentration is increased, TG2 is rapidly activated into an open conformation.

Most biochemical and cellular studies of TG2 used a particular variant with a glycine sequence at position 224 (TG2 G224). However, it has been suggested that the natural variant of TG2 may possess valine, instead of glycine, at position 224 (TG2 G224V) [25]. Human exon sequencing data in the various sequence databases, including NCBI, Ensembl, and ESP, showed a valine residue at position 224 of TG2, supporting the idea that the G224V form, rather than the G224 form, is the natural TG2 variant. In this study, we characterized the putative natural form of TG2, TG2 (G224V), and through a new crystal structure of TG2 (G224V), we revealed how TG2 (G224V) gained better stability and higher Ca2+-dependent activity than an artificial variant of TG2 (G224).

## Materials and methods

### Protein expression and purification

The TG2 (G224V) form was purified using a similar purification method previously described [21]. Briefly, full-length human TG2, corresponding to amino acids 1–687, cloned in pOKD homemade vector was used as a template for site-directed mutagenesis. Quick-change mutagenesis kit (Stratagene) was used according to the manufacturer’s protocols. Mutagenesis was then confirmed by sequencing. The G224V clone in pOKD vector was then transformed into BL21 (DE3) *E*. *coli* competent cells. Expression was induced by treating the bacteria with 0.125 mM isopropyl β-d-thiogalactopyranoside (IPTG) for 25 h at 18°C. Cells expressing TG2 (G224V) were pelleted by centrifugation, resuspended, and lysed by sonication in 50 ml lysis buffer (50 mM sodium-phosphate buffer at pH 7.5, 400 mM NaCl, 5 mM benzamidine, 1 mM 2-mercaptoethanol, 50 μM GTP, 1 mM PMSF, 0.5% (v/v) triton X-100 and 5 mM imidazole with protease inhibitor cocktail (Roche Applied Science, CA, USA)). The lysate was then centrifuged at 16,000 rpm for 30 min at 4°C, after which the supernatant fractions were applied to a gravity-flow column (BioRad) packed with Ni-NTA affinity resin (Qiagen). After washing the resin with 100 ml of washing buffer (50 mM sodium-phosphate buffer at pH 7.5, 400 mM NaCl, 5 mM benzamidine, 1 mM 2-mercaptoethanol, 50 μM GTP, 1 mM PMSF, 0.5% (v/v) triton X-100 and 5 mM imidazole), the bound TG2 (G224V) was eluted from the column, using elution buffer (50 mM HEPES buffer at pH 7.0, 100 mM NaCl, 50 μM GTP, 10% (v/v) glycerol and 300 mM imidazole). The eluted fractions were pooled and applied to a Superdex 200 gel filtration column (GE healthcare) that had been pre-equilibrated with a solution of 20 mM Tris at pH 8.0 and 150 mM NaCl. The purified TG2 (G224V) was subsequently applied to a mono Q ion-exchange column. Finally, the eluted TG2 (G224V), from ion-exchange chromatography, was pooled and applied to a Superdex 200 gel filtration column (GE healthcare) that had been pre-equilibrated with a solution of 20 mM Tris at pH 8.0 and 150 mM NaCl. The purified TG2 (G224V) was collected and concentrated to 10 mg ml^-1^. The final protein sample was then confirmed to contain TG2 (G224V) by SDS-PAGE.

### Multi angle light scattering (MALS)

The absolute molar mass of TG2 (G224V) was determined with multi angle light scattering (MALS). Briefly, the target protein was loaded onto a Superdex 200 HR 10/30 gel-filtration column (GE Healthcare) that had been pre-equilibrated in a buffer containing 20 mM Tris-HCl pH 8.0 and 150 mM NaCl. The ÄKTA chromatography system (GE Healthcare) was coupled to a MALS detector (mini-DAWN treos) and a refractive index detector (Optilab DSP) (Wyatt Technology).

### Transamidation (TG) activity assay

TG2 activity was determined by measuring the incorporation of biotinylated pentylamine (BP, Pierce, Rockford, IL) into *N*,*N′*-dimethylcasein (Sigma, St. Louis, MO), as previously described with the following modifications [26]. Briefly, 10 nM of purified human TG2 (G224) or TG2 (G224V) was incubated with the substrate solution (100 μg/mL of *N*,*N′*-dimethylcasein, 100 μM BP, 50 mM Tris-HCl, pH 7.5, 5 mM dithiothreitol, 150 mM NaCl, and 1% Triton X-100) at various concentrations of CaCl_2_ for 1 h at 37°C. The reaction was stopped by adding 20 mM EDTA. 100 μL of the reaction mixtures was coated to each well of a 96 well microtiter plate (Nunc, Roskilde, Denmark) by incubating for 18 h at 4°C. After subsequent incubation with 200 μL of 5% BSA in PBS for 2 h at room temperature, the wells were washed four times with 200 μL of 1% BSA in PBS containing 0.1% Tween 20. BP, incorporated into *N*,*N′*-dimethylcasein, was probed using horse-radish peroxidase-conjugated streptavidin (Thermo Fisher Scientific, Carlsbad, CA), followed by the performance of the coloring reaction using *О*–phenylenediamine dihydrochloride. The reaction was stopped by adding 1 M H_2_SO_4,_ and the absorbance at 492 nm was measured with a microplate spectrophotometer (Molecular Devices, Sunnyvale, CA).

### Isothermal titration calorimetry (ITC)

A Nano ITC (TA Instruments) was used for the isothermal titration calorimetry experiments. Two variants of TG2, TG2 (G224V) and TG2 (G224) were dialyzed against PBS buffer with 0.5. mM EDTA, and Ca^2+^ was dissolved in the same buffer to minimize the heat of dilution values. Prior to titration, the protein samples and Ca^2+^ were centrifuged at 13,000 rpm at 4°C for 5 min to remove all precipitants. For incremental injection into ITC, 2 μl of 1 mM Ca^2+^ was injected into a sample cell containing 180 μl of either TG2 (G224V) or TG2 (G224) at a concentration of ~20 μM. All titrations were carried out at 15°C with 20 injections at 160 s intervals. The area under each titration peak was integrated, plotted against the number of injections, and fitted with a one-site independent binding model, using the software provided by TA instruments. Experimental data were subtracted from appropriate baselines acquired by injecting Ca^2+^ into the buffer, without the protein samples.

### Spontaneous degradation assay

Each 5 μl of 1 mg/ml protein samples of TG2 (G224) and TG2 (G224V) was incubated at room temperature for 12 h. The samples were applied to SDS-PAGE and the degradation pattern was analyzed with Coomassie blue staining.

### Time-dependent aggregation assay

To compare the stability of TG2 (G224) and TG2 (G224V), we performed an aggregation assay. Both purified proteins were incubated at 25°C and 4°C for 24 h. The turbidity of each sample was directly measured, based on the optical density, at 600 nm using a spectrophotometer (Beckman).

### Crystallization and data collection

The crystallization conditions were initially screened at 20°C with the hanging-drop vapor-diffusion method. Similar to previously employed conditions, crystals were grown on plates by equilibrating a mixture containing 1 μl protein solution, 1 μl of reservoir solution (20 mM MES at pH 6.2, 150 mM sodium chloride, 5 mM MgCl_2_, 4% PEG 3350, 5 mM DTT), and 20% glycerol against 0.4 ml of the reservoir solution. A 3.2 Å native diffraction data set was collected from a single crystal on beamline 5C at the Pohang Accelerator Laboratory (PAL), South Korea. The data sets were indexed and processed with HKL2000.

### Structure determination and analysis

The structure was determined by the molecular replacement phasing method, using *Phaser* [27]. One chain of the previously solved structure of GTP-bound TG2 (PDB code: 4PYG) [21] was used as a search model. Model building and refinement were conducted by COOT [28] and REFMAC5 [29], respectively. The geometry was inspected using PROCHECK and was found to be reasonable. All molecular figures were generated using the program PyMol [30].

### GTPase activity assay

GTPase activity of TG2 was determined by ATPase/GTPase Activity Assay Kit (Sigma St. Louis, MO) according to the manufacturer’s instructions with the following modification. Various amounts (2, 4, and 8 μg) of purified human TG2 (G224) or TG2 (G224V) were incubated in 40 μL of reaction solution (20 mM Tris, pH7.5, 40 mM NaCl, 4 mM MgAc2, 0.5 mM EDTA, 23.75% Glycerol, 0.525 mM DTT, and 1 mM GTP) for 2 h at 37°C. The reaction was stopped by adding 200 μL of malachite green reagent solution, followed by incubation for an additional 30 min at room temperature to generate the colorimetric product. The absorbance at 620 nm was measured on a microplate spectrophotometer (Molecular Devices, Sunnyvale, CA). The amount of free phosphate liberated by TG2 was calculated by the values obtained from a phosphate standard solution which was diluted in the reaction solution.

### Protein data bank accession code

The coordinates and structure factors have been deposited in the Protein Data Bank (PDB) with the PDBid of 6A8P.

## Results

### TG2 (G224V) is more stable and has higher Ca^2+^-dependent activity than TG2 (G224)

**Fig 1:**
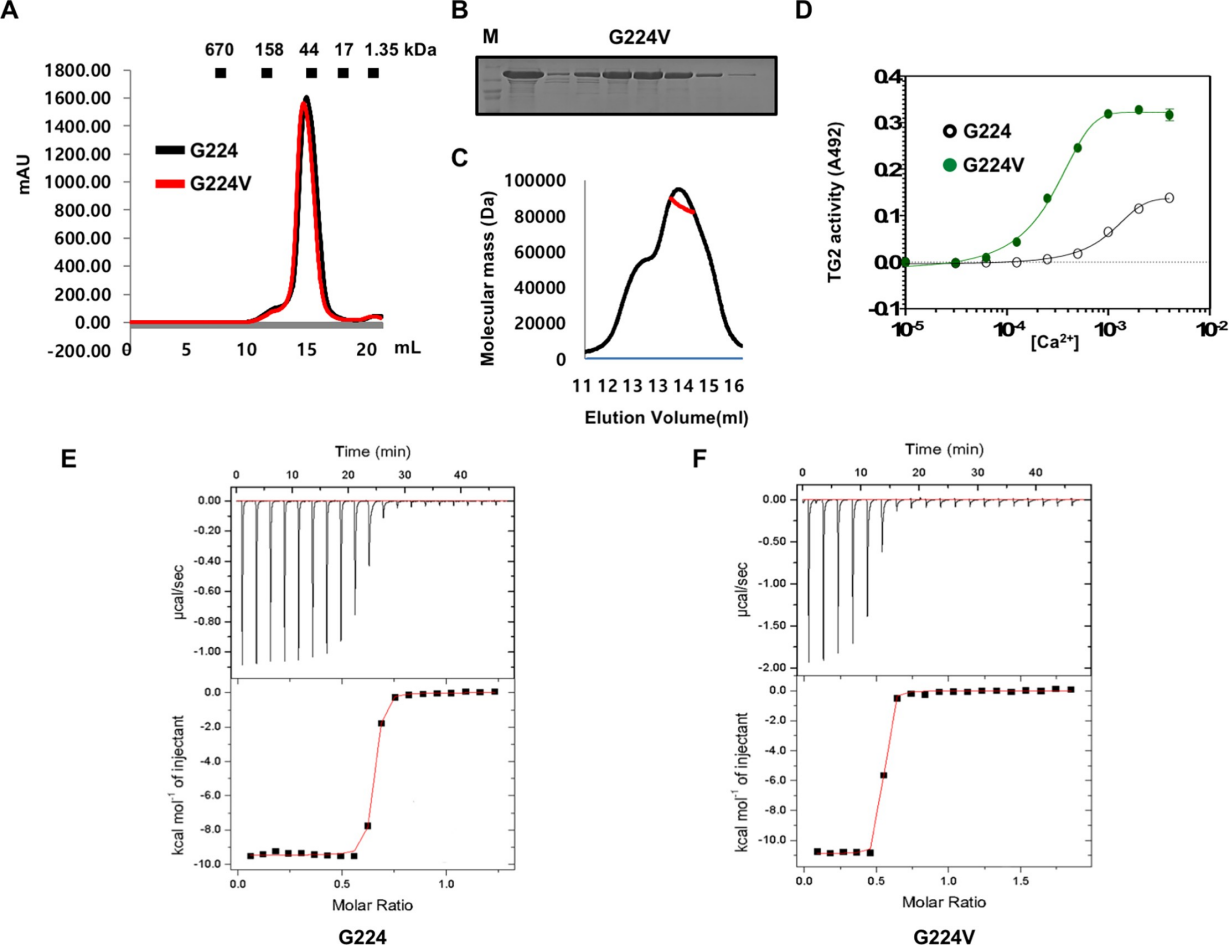
Characterization of TG2 (G224V). (A) Size-exclusion chromatograms of TG2 (G224V) and TG2 (G224). Profiles obtained from two TG2 variants in buffer containing 20 m*M* Tris-HCl at pH 8.0 and 150 m*M* NaCl are shown. (B) SDS-PAGE shows the peak fractions from the size-exclusion chromatography of TG2 (G224V). Protein size markers are indicated. (C) Multi-angle light scattering (MALS) measurement of TG2 (G224V). The x-axis and y-axis indicate the elution volume and molecular mass, respectively. (D) The effect of various calcium concentrations for transamidation activity of TG2 (G224) and TG2 (G224V). (E and F) Comparison of Ca^2+^ binding affinity between two TG2 variants, G224V (E) and G224 (F), via ITC.

**Fig 2:**
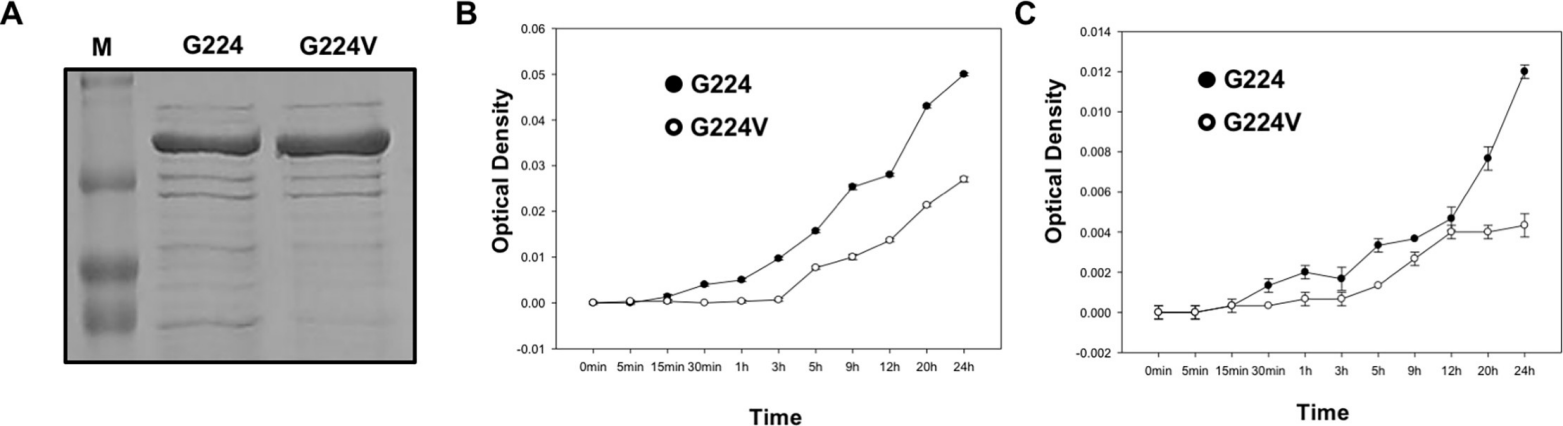
Stability comparison between TG2 (G224) and TG2 (G224V). (A) Spontaneous proteolysis assay with SDS-PAGE. M indicates Marker and two TG2 variants are indicated above the proper lanes. (B and C) Stability comparison between TG2 (G224V) and TG2 (G224). Same stability experiments were performed at two different temperatures, 25°C (B) and 4°C (C).

It is known that the natural TG2 form contains valine, instead of glycine, at position 224. To understand the effect of G224V mutation on TG2, we characterized, solved the structure of TG2 (G224V), and compared it with the TG2 (G224) form. For this study, TG2 (G224) in pOKD homemade vector was mutated to valine at glycine position 224 to generate the TG2 (G224V) form. The new mutated TG2, TG2 (G224V) form, was expressed and purified using the method used for the purification of the TG2 (G224) form. Because previous biochemical and structural studies showed that TG2 could exist as a monomer or dimer in solution, the stoichiometry of the TG2 (G224V) form was analyzed with size-exclusion chromatography. Both forms of TG2 came out around 15 ml in size-exclusion chromatography (Fig 1A), indicating that both forms exist as a monomer in solution and mutating glycine at position 224 to valine did not have any effect on the stoichiometry of TG2. The purity of the TG2 (G224V) form was similar to that of the TG2 (G224) form (Fig 2B). To obtain the accurate molecular weight of TG2 (G224V) in solution, MALS experiment was performed. The theoretically calculated molecular weight of the monomeric TG2 (G224V), including the C-terminal His-tag and GTP, was 78.90 kDa, and the experimental molecular weight from MALS was 80.24 kDa (1.82% fitting error), with a polydispersity of 1.2 (Fig 1C). Based on our analysis with size-exclusion chromatography and MALS, we concluded that the TG2 (G224V) form, like the TG2 (G224) form, exists as a monomer in solution, indicating that mutation from glycine to valine at position 224 of TG2 did not alter the monomeric state of TG2. Next, transamidation activity of both forms was analyzed at various Ca^2+^ concentrations using a monoamine 5-biotinamido-pentylamine (BP) as an amine substrate. As shown Fig 1D, the maximum activity of TG2 (G224V) was about 2.3-fold higher than that of TG2 (G224). Moreover, TG2 (G224V) was approximately 4.4-fold more sensitive to Ca^2+^ than TG2 (G224), with Ca^2+^ EC_50_ values of 0.211±0.038 mM and 0.929±0.06 mM, respectively. ITC was used to analyze the Ca^2+^- binding affinity of the two TG2 variants. In disagreement with previous binding analyses [25], which reported a Kd of ~0.5 μM for TG2 (G224) with 0.5 mM Ca^2+^, the current ITC analysis showed that TG2 (G224) possessed a Kd of ~0.02 mM with 0.5 mM Ca^2+^ (Fig 1E). For the G224V variant, Kd was calculated to be 0.01 mM in the presence of 0.5 mM Ca^2+^, indicating that the G224V variant showed a better affinity for Ca^2+^, approximately two-fold (Fig 1F). Although this result differs from that of previous reports, it still shows that TG2 (G224V) has a better affinity for Ca^2+^ [25]. Although complicated Ca^2+^-binding sites on TG2, at least, six binding sites with one strong and five weak interaction sites for controlling the activity of TG2, have been identified [31], this binding study indicates that both variants have similar affinity for the strong Ca^2+^-binding site at low Ca^2+^ concentrations.

To compare the stability of the two TG2 forms, spontaneous degradation assay and time-dependent aggregation assay were conducted. After 12 h of incubation at room temperature, both TG2 (G224) and TG2 (G224V) were loaded on SDS-PAGE. The stained gel indicated that TG2 (G224) was degraded on the gel more than G224V (Fig 2A). Time-dependent aggregation assay results showed that TG2 (G224) was more prone to aggregation and precipitation than TG2 (G224V), either at 25°C (Fig 2B) or 4°C (Fig 2C). Stability analysis of the two forms showed that TG2 gains stability by mutating glycine to valine at position 224.

### Structure of TG2 (G224V)

**Fig 3:**
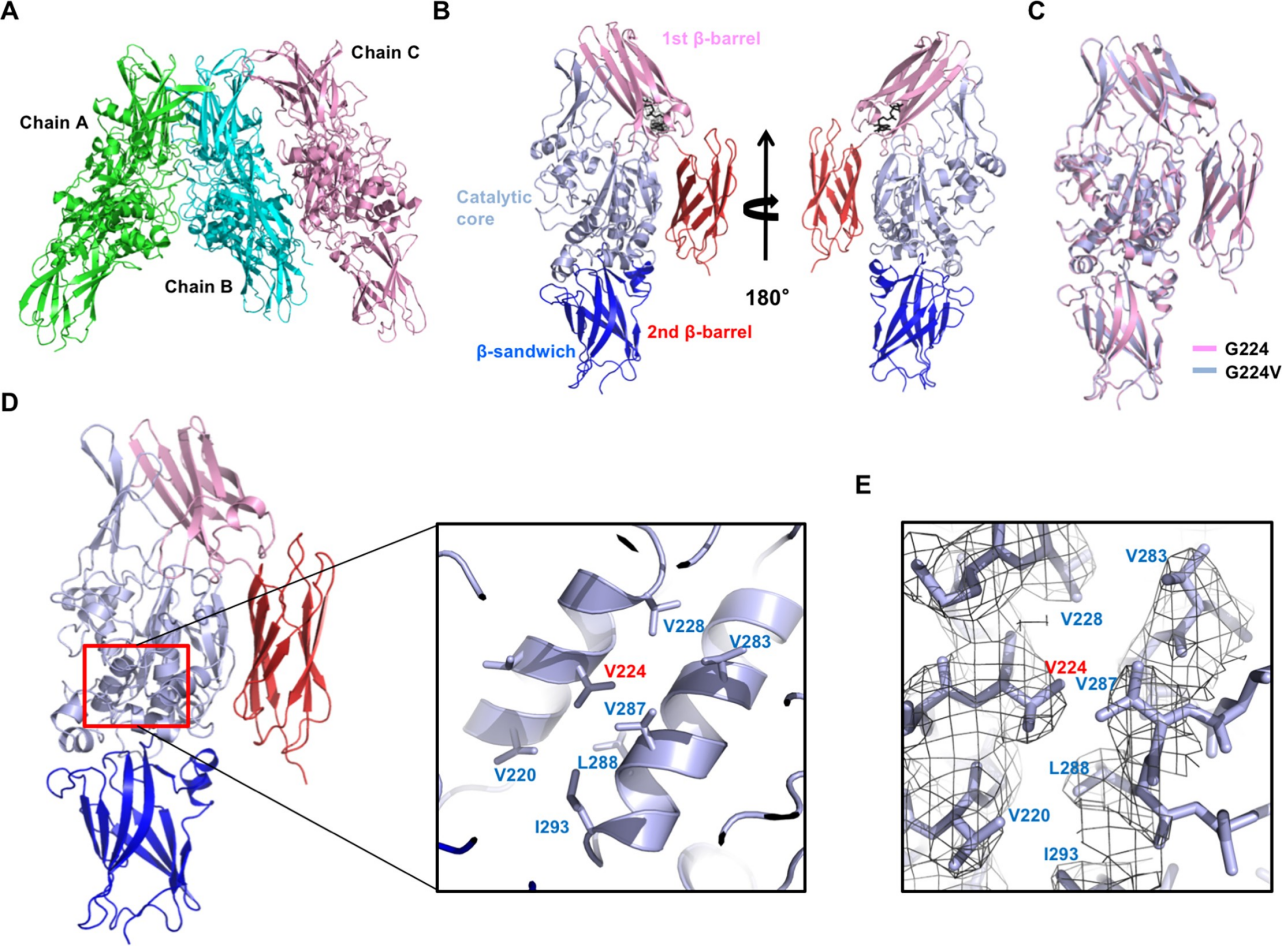
Crystal structure of TG2 (G224V) form in complex with GTP. (A) Ribbon diagram of the structure of the TG2 (G224V) form in complex with GTP. Three molecules in the asymmetric unit are shown (Chain A, Chain B, and Chain C). (B) Domain boundary and GTP binding site on the TG2 (G224V) form. Each domain is indicated by a different color scheme, blue for (β-sandwich), light blue for (catalytic core), pink for (1st β-barrel), and red for (2nd β-barrel). Bound GTP at 1st β-barrel domain is shown with black stick model. (C) Overall structural comparison of TG2 (G224V) with TG2 (G224) by superimposition. (D) The location of amino acid position 224 on TG2. Red box indicates the G224-containing region. The region is magnified and shown at the right panel. Mutated G224 to V (G224V) was shown with neighboring hydrophobic residues that can form hydrophobic cluster with V224. (E) An omit density map contoured at the 1-σ level around V224.

Purified TG2 (G224V) was crystallized under conditions similar to that used for the crystallization of TG2 (G224). The 2.5 Å crystal structure of TG2 (G224V) was solved and refined to an *R*_work_ of 23.6% and an *R*_free_ of 25.6%. The data collection and refinement statistics are summarized in Table 1. There were three monomers in the asymmetric unit, chain A, chain B, and chain C (Fig 3A). We did not build models for the loops because of the poor electron density map. Residue L688 is an extra residue from the cloning construct. All three chains were identical and contained GTP (Fig 3B), which is similar to that of the recently solved GTP-bound form of TG2 (G224) [21]. The structure of the TG2 (G224V) variant showed that the overall structure is similar to the previously reported structure of GDP-, ATP-, GTP- bound closed form of TG2. The root mean square deviations (RMSD) between the TG2 (G224) and TG2 (G224V) variants was 0.51 Å (Fig 3C), indicating that replacement of G224 with valine did not have any effect on the overall structure of TG2. The G224 site on the structure was carefully analyzed and we found that the amino acid residue at site 224 is a critical residue for the formation of hydrophobic clusters with neighboring hydrophobic residues, including V287 and L288, which are located on active site cysteine (C277)-containing ɑ-helix in the catalytic core (Fig 3D). We used RING2.0 web server [32] and manual distance measurement via Pymol [33] for analysis the interaction of the site 224. Although we double-checked the glycine to valine mutation at position 224 with DNA sequencing, we confirmed the V224 replacement with the electron density of the structure. A typical valine density with a small bulb shape in the density map clearly indicated that TG2 (G224) was mutated to TG2 (G224V) (Fig 3E).

**Table 1:** Crystallographic statistics.

Data collection	Native
Space group	*C222*_*1*_
Cell dimensions	
*a*, *b*, *c*	133.19Å, 216.31Å, 166.04Å
Resolution	50–2.5Å
^*a*^*R*_sym_	13.2% (141.1%)
^*a*^Mean I/σ(I)	19.8 (1.8)
^*a*^Completeness	99.6% (99.9%)
^*a*^Redundancy	13.0 (12.5)
**Refinement**
Resolution	50–2.5Å
No. reflections used	77,951
*R*_work_/*R*_free_	23.6%/25.8%
No. atoms	
Protein	15,930
Water and other small molecules	96
Average B-factors	52.5 Å^2^
R.M.S deviations	
Bond lengths	0.007Å
Bond angles	1.24°
Ramanchandran plot	
Most favored regions	97.0%
Additional allowed regions	2.8%

^a^Highest resolution shell is shown in parenthesis.

### Structural comparison of TG2 (G224V) with TG2 (G224)- active site

**Fig 4:**
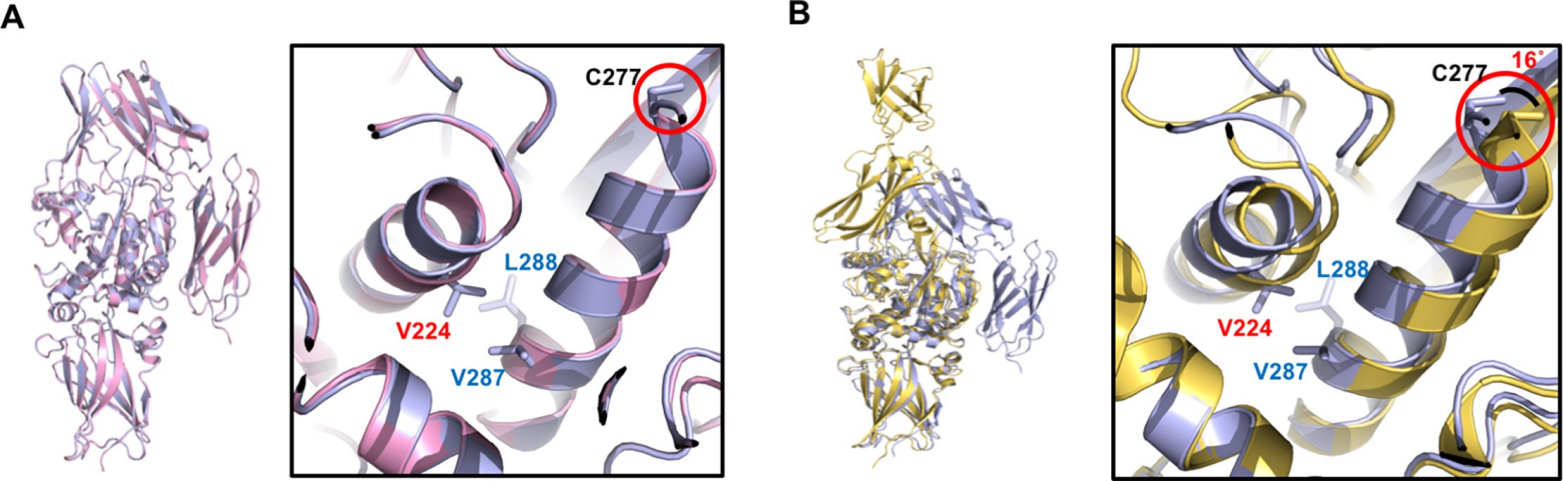
Structural comparison between two variants of TG2, G224V form and G224 form. (A) Superposition of the structure of the TG2 (G224V) form (light blue color) with a known structure of TG2 (G224) form (pink color). Red-circle indicates active site cysteine (C277), which is a critical residue for the cross-linking activity of TG2. (B) Superposition of the structure of the TG2 (G224V) form (light blue color) with the structure of the open TG2 (G224) form (yellow color). 16° movement of active site cysteine (C277) when TG2 (G224) was activated to the open form is shown by red-circle.

To analyze the structural alteration resulting from the introduction of mutation (G224 to V224) into TG2, two structures, previously solved TG2 (G224) and currently solved TG2 (G224V), were superimposed. As shown in Fig 4A, the structure of TG2 (G224V) was almost identical with that of TG2 (G224) closed form, showing a 0.51 Å root mean square deviation (RMSD). When the structure of TG2 (G224V) was superposed with the open form of TG2 (G224), a structural change was detected around amino acid position 224. The active site cysteine (C277)-containing ɑ-helix, which is stabilized by the formation of a hydrophobic cluster with neighboring V224, was shifted to the opposite direction by 16°, especially the active site cysteine region (Fig 4B). Since the active site cysteine (C277)-containing ɑ-helix, whose location may be important for TG2 activity, is interacted with a neighboring intramolecular ɑ-helix, which contains residue 224, replacement of G with V will have an effect on the transamidase activity of TG2. Decreased activity of G224 may be due to the higher chance of location shift on active site cysteine (C277) because of the loss of the hydrophobic cluster anchored by V224.

### Structural comparison of TG2 (G224V) with TG2 (G224)-calcium binding site

**Fig 5:**
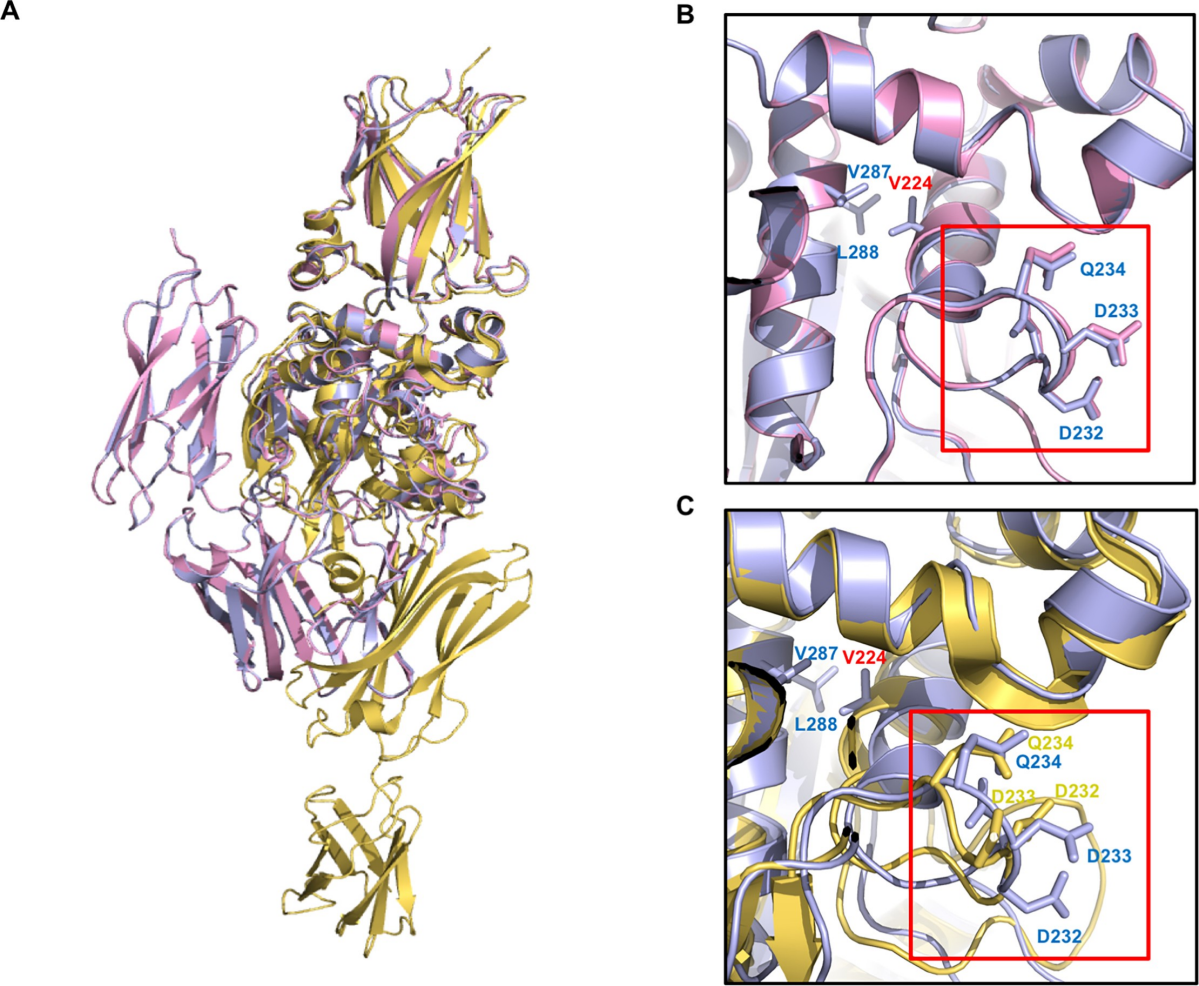
Comparison of representative Ca^2+^ binding site in the two TG2 variants. (A) Superposition of the structure of the TG2 (G224V) form (light blue color) with known structures of TG2 (G224) form (pink color) and open form of TG2 (G224) (yellow color). (B and C) Pairwise structural comparison between the structure of the TG2 (G224V) form (light blue color) with the structure of the TG2 (G224) form (pink color) (B) and with the structure of the open TG2 (G224) form (yellow color) (C). Representative Ca^2+^ binding site is indicated by a red-box.

Because the activity of TG2 has to be tightly regulated, many activity regulators have been identified [34]. Among them, the main TG2 activity regulators are Ca^2+^ and GTP. The activity of TG2 is positively regulated by Ca^2+^ and negatively regulated by nucleotides such as GTP, GDP, and ATP [17]. The Ca^2+^-mediated activation mechanism is still not well characterized in TG2 without available complex structures, whereas the structural information of Ca^2+^-bound TG3 subfamily is available [35]. In the case of TG3, three Ca^2+^ binding site, located around catalytic core domain, were identified in the structure. Upon Ca^2+^ binding and activation of TG3, it undergoes structural changes at around flexible surface loop in the B-sandwich domain and the catalytic core domain in the vicinity of each of the Ca2+-binding sites [35]. Ca^2+^-binding sites of TG2 have been characterized by mutational studies without structural information [31]. Based on this, at least, six Ca^2+^-binding sites on TG2, one strong and five weak interaction sites, have been identified. One strong binding site was assigned from reside 228 ~ 236 and five weak interaction sites include residue 396 ~ 458, 306 ~ 312, 328 ~ 330, 151 ~ 158, and 433 ~ 438 of TG2. Our characterization of TG2 (G224V) showed that TG2 (G224V) activity was much higher than that of TG2 (G224) in the presence of proper Ca^2+^ concentrations, whereas the Ca^2+^-binding affinity was almost the same in the presence of low Ca^2+^ concentration in both variants. Because of these observations, the structures of the main and high affinity Ca^2+^-binding site of both variants, which is considered as the primary Ca^2+^-binding site and corresponds to V228~V236, were compared by structural alignment (Fig 5A, 5B and 5C). In addition, G224 is located on the α-helix directly connected to the primary Ca^2+^-binding loop, which means that G224V mutation can affect the structure of the primary Ca^2+^-binding site and consequently affect the activity of TG2. Superposition analysis showed that the structures of the high affinity Ca^2+^-binding site of both variants were exactly superposed (Fig 5B), indicating that mutation from G to V did not have any effect on the structure of the primary Ca^2+^-binding site on TG2. However, compared with the open TG2 (G224) form, a huge structural alteration in the primary Ca^2+^-binding loop was detected (Fig 5C), indicating that G224V mutation in the α-helix directly connected to the primary Ca^2+^-binding loop can affect both Ca^2+^-binding and activity of the opened and active TG2 form. Hydrophobic patch formation, through the newly introduced G224V, may lead to fixation of the G224-containing α-helix and to some extent affect both Ca^2+^-binding and TG2 activity. This may indicate that, when TG2 is activated and transformed to the open form, proper Ca^2+^-binding at the proper position of the primary Ca^2+^-binding site is important for the full activity of TG2.

## Discussion

Although most biochemical and cellular studies have been conducted with the TG2 (G224) form, it has been suggested that the actual sequence at position 224 on TG2 may be valine, and not glycine. Human exon sequencing data deposited in the NCBI, Ensembl, and ESP, showed that a valine residue is found at position 224 of TG2, supported the idea that the G224V form, rather than the G224 form, is the natural variant of TG2. Although it has been shown that the TG2 (G224V) form is more stable and has a higher activity in the presence of Ca^2+^ [25], no structural information is available to show how the mutation from G to V confers this ability on the TG2 (G224V) form. In this study, we characterized the putative natural form of TG2, TG2 (G224V), and through a new crystal structure of TG2 (G224V), we revealed how TG2 (G224V) gained stability and higher Ca^2+^-dependent activity than an artificial variant of TG2 (G224).

According to our characterization, TG2 (G224V) exists as a monomer in solution, similar to TG2 (G224), indicating that mutation from glycine to valine at position 224 did not alter the stoichiometry of TG2. ITC measurement showed that the Ca^2+^- binding affinity of the two TG2 variants is almost the same, approximately two-fold difference, exhibiting a Kd of 0.01 ~ 0.02 μM, with a single site interaction mode. This result did not match previous Ca^2+^ affinity measurements because TG2 (G224) showed a Kd of around 0.3~0.8 μM. Because there are many Ca^2+^-binding sites on TG2 with various affinities, it is not surprising that various Kd values may be calculated under different conditions. Although Ca^2+^ affinity differed by two-fold, Ca^2+^-dependent activity was highly different between the two variants. The activity of TG2 (G224V) was approximately 3-fold higher than that of TG2 (G224). In addition, we also observed a difference in stability between the two variants. The G224V form was much more stable than the G224 form, regardless of temperature changes.

Because the putative natural variant TG2 (G224V) showed a higher activity and stability than the artificial variant TG2 (G224), we tried to understand how the TG2 (G224V) form gained higher activity and stability by solving the crystal structure of TG2 (G224V) and comparing it with the structure of a previously solved TG2 (G224). The structure showed that G224 is positioned on an ɑ-helix located at the opposite side of an ɑ-helix containing active site cysteine (C277). Since active site cysteine (C277)-containing ɑ-helix is shifted to the opposite direction at 16° when TG2 is activated to the open form, valine substitution at position 224, which can form a stable hydrophobic cluster with hydrophobic residues of active site cysteine (C277)-containing ɑ-helix, can affect the location of active site cysteine and consequently the TG2 activity. The primary Ca^2+^-binding site, which has a high Ca^2+^-binding affinity and is formed by a loop corresponding to amino acids V228~V236, is located next to a G224-containing α-helix. Because we detected huge structural changes in the primary Ca^2+^-binding site when TG2 was activated, G224V mutation may have an effect on the structure of the primary Ca^2+^-binding site, especially when it is activated. Based on these results, we conclude that hydrophobic patch formation, via the newly introduced G224V, may lead to fixation between G224-containing α-helix, which is connected to the Ca^2+^-binding site loop, and active site cysteine (C277)-containing α-helix. This structural connection can affect the movement of active site cysteine and the formation of Ca^2+^-binding site when TG2 is activated to the extended form, which may be critical for the full activation of TG2.

**Fig 6:**
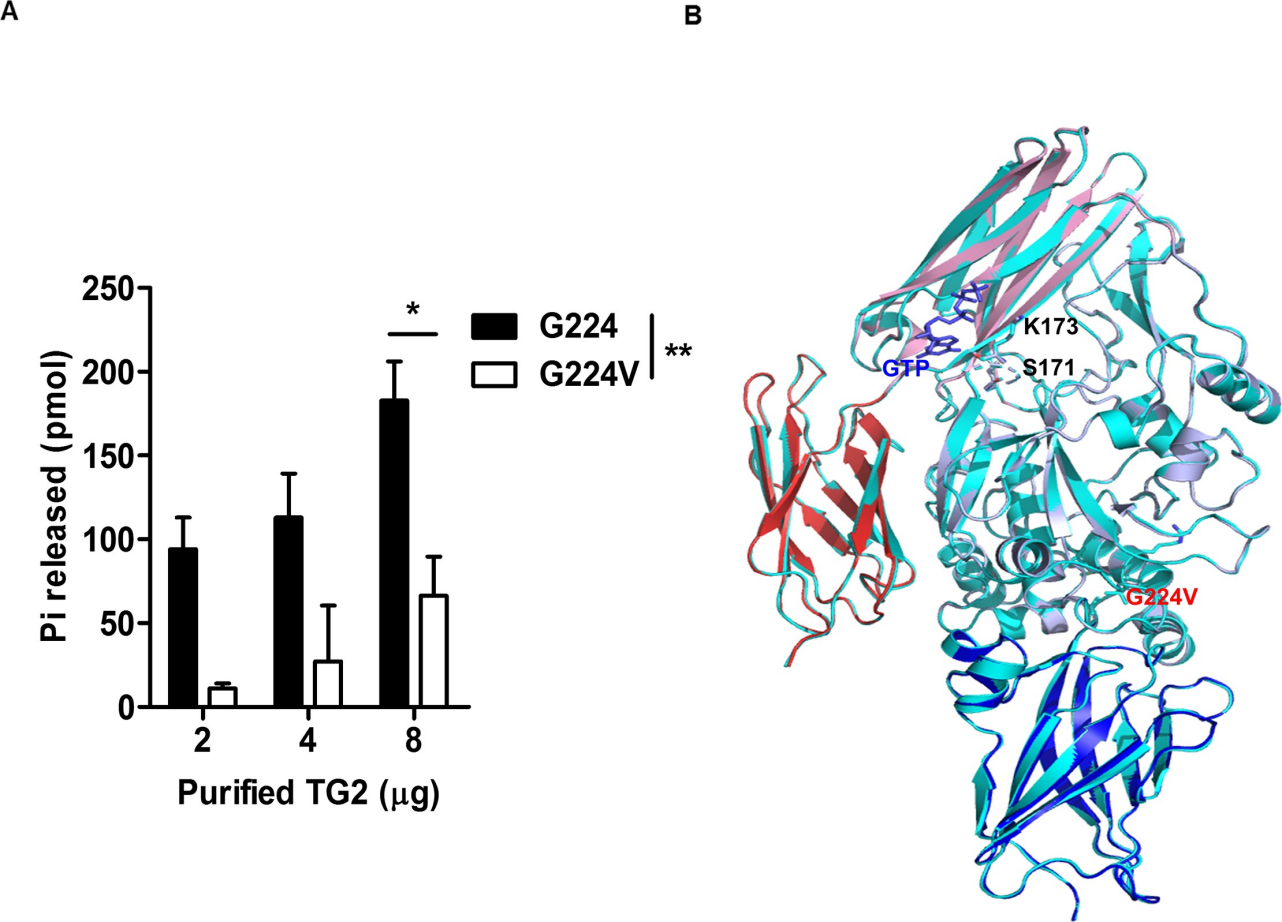
Comparison of GTPase activity of two variants. (A) GTPase activity assay. Various amounts of purified human TG2 (G224) or TG2 (G224V) were incubated in reaction solution for 2 h at 37°C. The reaction was stopped and the solution was incubated for an additional 30 min at room temperature to generate the colorimetric product. The absorbance at 620 nm was measured on a microplate spectrophotometer to analysis the amount of free phosphate liberated by TG2. (B) Superposition of the structure of the TG2 (G224V) form (mixed color) with known structures of TG2 (G224) form (cyan color). Bound GTP is shown with blue stick model. Mutated G224V is shown with red. Important residues for GTP binding are labelled with black color.

GTPase activity is one of the multi-function of TG2. To checked whether two different variants have any different GTPase activity, we compared GTP hydrolysis activity. Interestingly, artificial variant TG2 (G224) has approximately four times higher activity than natural variant TG2 (G224V) (Fig 6A). Because GTP binding region is structurally conserved in between two variants (Fig 6B), it is hard to understand how TG2 (G224) variant get higher GTPase activity with current model. This should be studied in the future.
